# SMITE: A toolbox for creating Psychophysics Toolbox and PsychoPy experiments with SMI eye trackers

**DOI:** 10.3758/s13428-019-01226-0

**Published:** 2019-04-01

**Authors:** Diederick C. Niehorster, Marcus Nyström

**Affiliations:** 1 Lund University Humanities Lab, Lund University, Lund, Sweden; 2 Department of Psychology, Lund University, Lund, Sweden

**Keywords:** Eye tracking, Eye movements, Stimulus creation, Equipment interface, SMI

## Abstract

We present SMITE, a toolbox for the measurement of eye movements using eye trackers manufactured by SMI GmbH. The toolbox provides a wrapper around the iViewX SDK provided by SMI, allowing simple integration of SMI eye trackers into Psychophysics Toolbox and PsychoPy programs. The toolbox provides a graphical interface for participant setup and calibration that is implemented natively in Psychophysics Toolbox and PsychoPy drawing commands, as well as providing several convenience features for, inter alia, creating gaze-contingent experiments and working with two-computer setups. Given that SMI GmbH and its support department have closed down, it is expected that this toolbox will provide owners of SMI eye trackers with an important new way to continue to create experiments with their systems. The eye trackers supported by this toolbox are the SMI HiSpeed 1250, SMI RED systems, SMI RED-m, SMI RED250mobile, and SMI REDn.

## Introduction

Software packages that provide an easy interface between low-level manufacturer software development kits (SDKs) for measuring eye movements and visual stimulation programs written in high-level programming languages have been around for a long time (e.g., the EyeLink Toolbox; Cornelissen, Peters, & Palmer, 2002) and have facilitated significant research using eye movement recordings. These packages provided by researchers and eye-tracker manufacturers provide an interface to a specific eye tracker, but high-quality system-specific packages are not available for eye trackers from all manufacturers. Recent years, however, have seen the emergence of eye-tracker interface packages that offer a generic programming interface to eye trackers of multiple manufacturers, partially solving this problem. Although these packages, such as PyGaze (Dalmaijer, Mathôt, & Van der Stigchel, 2014) and the ioHub library that is part of PsychoPy, support many commonly used eye trackers, the ability to use multiple eye trackers with the same experiment program comes with the drawback that the generic interface for these packages limits access to the advanced setup and operation capabilities of some of the supported eye trackers.

This situation means that eye trackers from some manufacturers—importantly, those developed by SensoMotoric Instruments GmbH (SMI)—have ended up without a software interface that is both feature-rich and easy to integrate with visual stimulation programs written in high-level programming languages, such as MATLAB and Python. In this article, we therefore present a feature-rich software package, SMITE (SMIs Track Eyes), that allows easy integration of SMI eye trackers into visual stimulation programs written in MATLAB with Psychophysics Toolbox (PsychToolbox; Brainard, 1997; Kleiner, Brainard, & Pelli, 2007; Pelli, 1997) and in Python with PsychoPy (Peirce, 2007, 2009).

SMITE builds on the SMI iViewX SDK and provides participant setup and calibration screens implemented directly with PsychToolbox or PsychoPy drawing commands. SMITE provides an easy-to-use interface to SMI eye trackers and can be integrated into existing visual stimulation programs with only a handful of lines of code. SMITE, however, also supports an advanced interface to SMI eye trackers, providing access to all setup and operational options of the supported SMI eye trackers. SMITE is available from https://github.com/dcnieho/SMITE (MATLAB) and https://github.com/marcus-nystrom/SMITE (Python).

## SMI eye trackers

In its roughly 25-year existence, SMI manufactured a wide range of eye trackers that are still in active use in many labs. With this toolbox, we concentrate on the tower-mounted and remote eye trackers released by SMI in roughly the last 15 years. Specifically, SMITE supports most of the eye trackers supported by the SMI iViewX SDK—that is, the SMI HiSpeed 1250 and SMI RED systems, such as the RED500, SMI RED-m, SMI RED250mobile, and SMI REDn. Support for the SMI HED systems was not implemented in SMITE, because these trackers operate in a very different way from the systems we support, and we judged it unlikely that many of these systems are still in use. Support for the HiSpeed 240 could not be implemented, because recent versions of the SMI iViewX SDK do not appear to be compatible with it.

These eye trackers span a wide range of capabilities. Whereas the SMI HiSpeed systems are tower-mounted systems that do not allow for movement of the participant’s head, all the RED systems are remote eye-tracking devices that allow the participant to move within a frustum-shaped area called the *headbox* while eye-tracking data are recorded (but see Hessels, Cornelissen, Kemner, & Hooge, 2015; Niehorster, Cornelissen, Holmqvist, Hooge, & Hessels, 2018). In this article, these remote eye trackers that allow participant movement will be referred to as “the RED systems,” whereas the tower-mounted eye tracker will be referred to as “the HiSpeed system.” Amongst other differences, the supported SMI systems vary in their sampling frequencies (ranging from 60 to 1250 Hz), provide data with vastly different noise levels, and by default differ in whether they provide monocular data, binocular data from each eye independently, or data averaging the gaze positions of the two eyes. Data recorded with these trackers are stored in .idf files, a proprietary binary storage format.

## Implementation

Two parallel versions of SMITE have been developed, one written as a native MATLAB class and using a MATLAB extension (MEX) file, and one written as a native Python class that is compatible with Python 2.7 and 3.6. For drawing the setup and calibration screens, the MATLAB version relies on the Psychophysics Toolbox extension (Brainard, 1997; Kleiner et al., 2007; Pelli, 1997), whereas the Python version relies on PsychoPy (Peirce, 2007, 2009). SMITE has been tested with SMI iViewX SDK version 4.4.26. The minimum version of the SMI iViewX SDK supported by SMITE is 4.4.10, since this was the first version to introduce 64-bit support. Older versions of the SMI iViewX SDK may work if only 32-bit support is desired, but this has not been tested. The reader can contact the authors for advice regarding where to find the SMI iViewX SDK.

The MATLAB and Python versions of SMITE have the same interface, but they follow the naming conventions of each language. That is, function names are camelCased for the MATLAB version and snake_cased for the Python version. The supported commands are listed in Table 1, along with a description of their functions. In this table, EThndl refers to the SMITE class instance created with the SMITE() constructor, through which you interact with the eye tracker. Note that all functions except SMITE.calibrate() are implemented in pure MATLAB or Python and do not carry Psychophysics Toolbox/PsychoPy or other libraries as dependencies.^1^

**Table 1.**
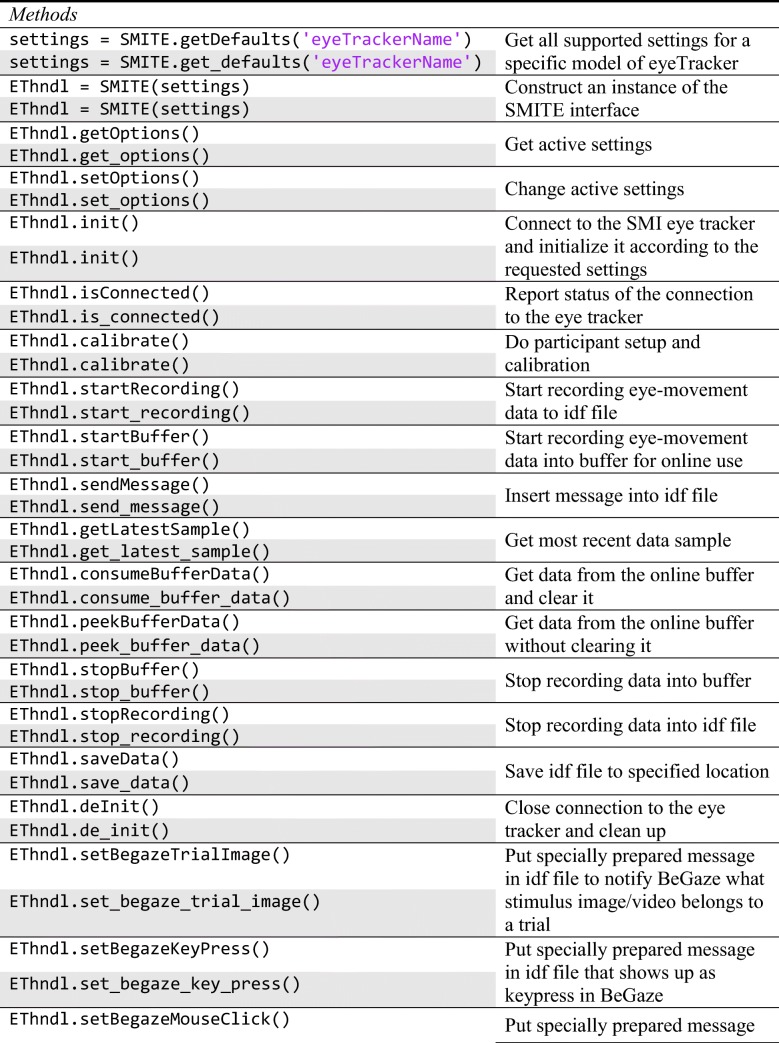

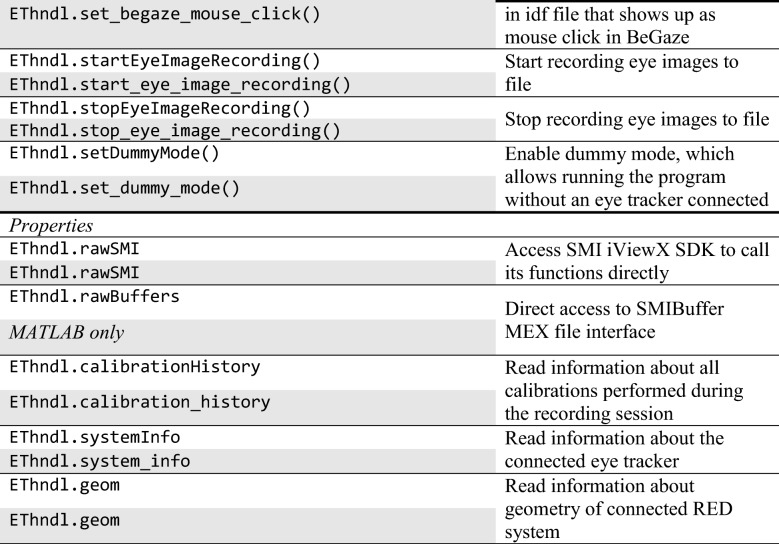
Overview of SMITE’s interface, along with a short description of the function of each method or property

MATLAB calls have a white background, and Python calls a gray background. Function input parameters and return values are not shown

### Participant setup

**Fig. 1:**
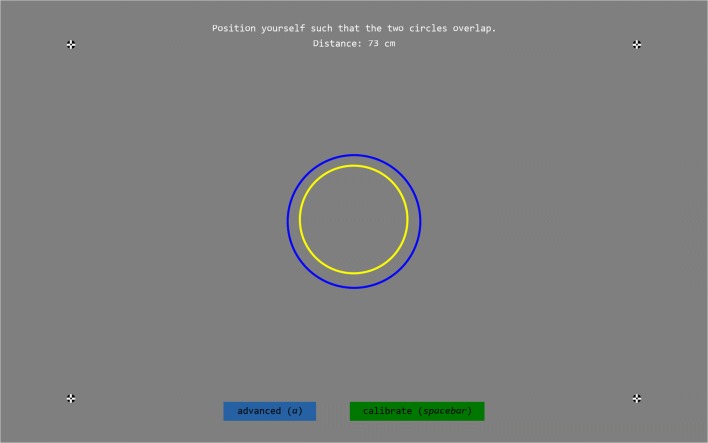
Basic graphical user interface for participant setup

By default, when calling SMITE.calibrate(), first a basic participant setup screen is shown. For the RED systems, this screen consists of two circles, a distance indicator and four fixation points in the corners (Fig. 1). The blue circle is a reference, indicating a participant who is sitting at the recommended or set viewing distance with their eyes horizontally and vertically centered in the headbox. The yellow circle indicates the participant’s current position in the headbox. Using this screen, a participant setup consists of asking the participant to sit such that the two circles overlap. Once this is achieved, we recommend asking the participant to look at the four fixation points in the corners, to ensure that data recording is stable across the screen.

**Fig. 2:**
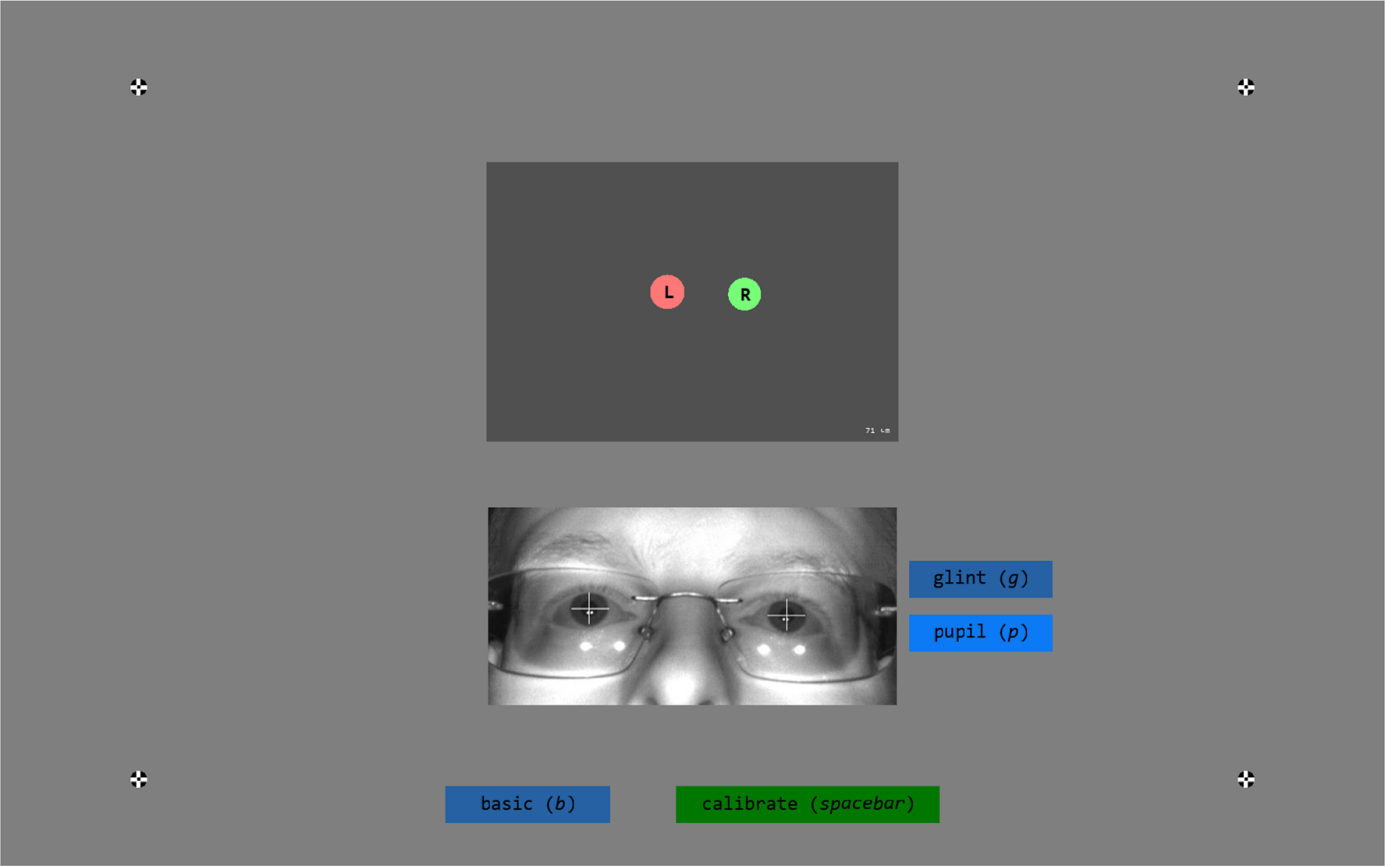
Advanced graphical user interface for participant setup. The glint and pupil toggle buttons enable or disable crosshairs that indicate where the pupil and glint were found in the eye tracker’s camera image

On the remote eye trackers, an advanced setup screen is also available (Fig. 2). This screen indicates where in the headbox the participant’s eyes are found and provides eye images^2^ to help troubleshoot the eye tracker and participant setup. For the HiSpeed systems, the setup screen always shows only the four fixation points, but eye images can be displayed by pressing the “a” key on the keyboard.

### Calibration and validation

By default, the calibration screen consists of a fixation point that minimizes drift and the microsaccade rate during fixation (ABC from Thaler, Schütz, Goodale, & Gegenfurtner, 2013; see the lower panel in their Fig. 1). The point jumps to present the calibration points in an order controlled by the SMI software. After calibration, the validation points are immediately shown in the same manner, so that to the participant the calibration and validation seem like one continuous stream of points to look at. This is done because there is no difference in task for the participant between the calibration and validation portions of setup.

To change what the calibration screen looks like, the programmer can provide their own calibration screen drawing function. This function will be called at the screen’s refresh rate with, as input, the numerical ID of the point to be drawn, its position, and a tick value that increases by 1 for each call. An example of drawing an animated calibration point is provided by the AnimatedCalibrationDisplay class that is provided with the toolbox.

The calibration can be restarted or interrupted at any time by pressing the “r” and “escape” keys, respectively. In the first case, the calibration starts over, and in the latter case, the calibration is cancelled and the script returns to the setup screen.

**Fig. 3:**
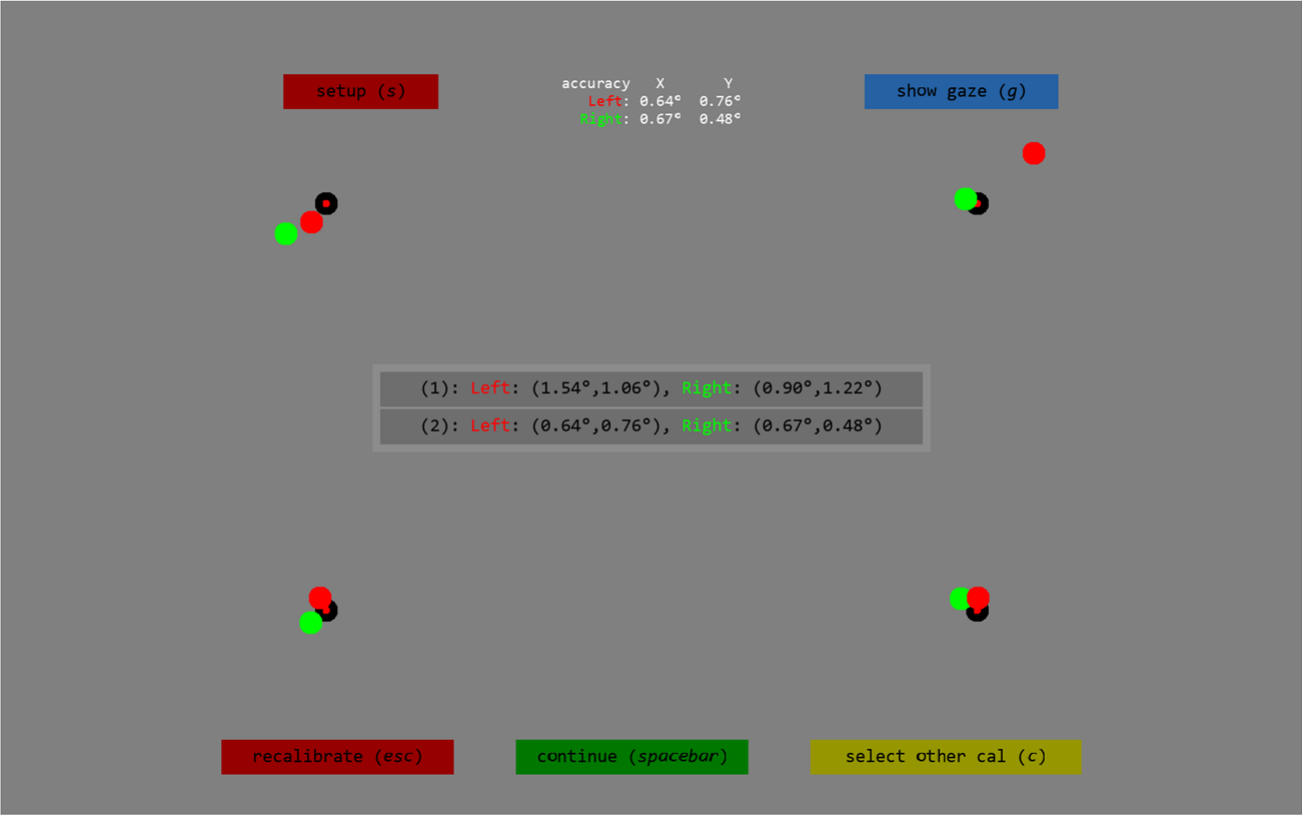
Validation result visualization. The *recalibrate* button starts a new calibration, and the *setup* button brings up the setup screen (see Fig. 1). The *show gaze* button toggles an online gaze display (not shown), and *select other cal* brings up a menu in the center of the screen that can be used to select other calibrations, if multiple calibrations have been performed

When SMITE.calibrate() completes after a calibration attempt, it returns information about the calibration and validation that were performed, including a visual and numerical representation of the accuracy (Fig. 3). It is also possible to inspect and select previous calibrations, as well as to visualize gaze online to further inspect calibration quality. These functionalities, respectively, enable the operator to select the best of multiple calibrations and to check whether inaccuracies unveiled by the validation output are due to inaccurate gaze toward the validation points or due to a bad calibration. Information about the accuracy of the calibration obtained through validation is written as a message into the idf data file, to ensure permanent storage.

It is highly recommended that the information about the calibration and validation that were performed be saved together with the eye movement data for each experimental run. This ensures that important information about data quality can be accessed during later analysis and that the quality of the calibration, as determined by the validation procedure, can be reported in the resulting scientific publication. Information about all calibrations that have been done during the lifetime of a SMITE class instance can be retrieved at any time using the property EThndl.calibrationHistory. In case the researcher wants to analyze the calibration and/or validation data in more detail, SMITE records sample data directly to the idf file throughout the calibration process.

The SMI RED250mobile and REDn introduced a “Smart Calibration” mode that automatically drops calibration points for which no eye-tracking data can be acquired, as well as providing the ability to manually skip calibration points. We have decided not to support this functionality in SMITE, because we think problems collecting data from a specific calibration point reflect an incorrect eye-tracker setup or an incompatible participant eye physiology and should not be dealt with in this manner. If data cannot be recorded from a specific location on the screen during calibration, data will also be lost at this location during the rest of the recording. This constitutes a problem that should be dealt with by correcting the setup or excluding the participant. It is, furthermore, unknown how the quality of the calibration will be affected by missing calibration points.

Calibration of the whole screen surface is often not required, because only part of the screen is being used to present stimuli. Because calibration using points at the corners of the screen may be more difficult than calibrating central regions of the screen, it is desirable to be able to calibrate only the part of the screen that will be used during the recording. SMITE therefore implements a set of options to scale and translate the grid of calibration points. This allows placing the grid such that it covers only part of the screen, while keeping the internal relations between the calibration points intact—which is probably important to achieving a valid calibration.

### SDK features not implemented

In SMITE, we have implemented a core set of functionality that we consider to be essential to many eye-tracking experiments and that provides the essentials for implementing advanced experimental paradigms, such as gaze-contingent displays. However, several capabilities are provided by the underlying SMI iViewX SDK that we have decided not to implement in SMITE. These remain accessible through the direct iViewX SDK access functionality of SMITE (see section “Direct access to SMI iViewX SDK” below).

The two features we have not implemented in SMITE are online event detection and area-of-interest (AOI) triggers. In both cases, we think neither an approach potentially provided by SMITE nor the algorithms implemented in the iViewX SDK would best suit most users who need such advanced tools, because any implementation would be geared to a particular use case that would likely differ from that of the user. A possible implementation of AOI triggers provided in SMITE might, for instance, force the user into an unnatural segmentation of their stimulus display, since only rectangle-shaped AOIs are supported, whereas a hypothetical event detector provided with SMITE might be tuned for saccade onset detection, but the user might instead need accurate identification of fixation onset. By using online data access through SMITE.getLatestSample(), SMITE.consumeBufferData(), and SMITE.peekBufferData(), users may implement these algorithms to their exact specifications or may use code available on the internet that best suits their needs.

### Dummy mode

To make it easier to develop a script using SMITE without access to the eye tracker, SMITE implements a dummy mode. When dummy mode is activated, by either calling SMITE.setDummyMode() or using the SMITEDummyMode() constructor directly, SMITE is put into a mode in which all calls are accepted, but it does not perform any action. If a SMITE call normally returns a value, in dummy mode SMITE will return [] in MATLAB, or None in Python. Exceptions to this rule are SMITE.getLatestSample(), SMITE.consumeBufferData(), and SMITE.peekBufferData(), which return the current position of the mouse cursor in order to make it easy to develop and test gaze-contingent code. The SMITE.rawSMI and SMITE.rawBuffers interfaces are not supported in dummy mode.

### File saving

Although the underlying SMI iViewX SDK allows overwriting of existing data files when calling its function to save recorded data to an .idf file, by default SMITE always returns an error when it attempts to save data when a file with the same name already exists. It is our opinion that overwriting data files should never happen in experimental practice, and that the cost of this occurring by mistake is too large for us to provide this flexibility in SMITE’s interface. Instead, SMITE.saveData() has an optional input argument that, when provided, instructs the function to automatically append versioning information to the filename provided by the user if a file with this name already exists. That is, if the user attempts to save data to the already existing file data.idf, the data will instead be saved to data_1.idf; if that file also already exists, the data will be saved to data_2.idf; and so forth. We recommend that user code generate the eventual filenames for the data files already before the experiment run starts and that it check whether these files already exist at that stage, so that naming mistakes can be corrected before any data are collected.

### BeGaze integration

Aside from eye trackers, SMI GmbH also provided Experiment Suite, a collection of stimulus program creation and data analysis applications. SMITE provides integration with SMI’s analysis application, BeGaze. This support has been tested with the last available version of BeGaze, included with Experiment Suite 3.7.104, but the SMITE is expected to work with a significant number of earlier versions of BeGaze, since the underlying interface has long been the same. To enable analysis of eye movement data in BeGaze, the .idf file record of the experiment needs to contain a specially formatted message for each trial informing BeGaze that a stimulus image or video was shown. These stimulus messages can be sent to the .idf file using SMITE.setBegazeTrialImage() and consist of the filename of the stimulus shown during the trial, which should end in one of the following extensions: .png, .jpg, .jpeg, .bmp, or .avi. Once such a stimulus message is sent to the file, data from that point onward will be associated with a new trial and the provided stimulus, until a new stimulus message is sent or the recording is stopped. To ensure that only the data of interest are imported by BeGaze for each trial, we therefore recommend the following sequence of calls for each trial:

1. SMITE.startRecording(). Start recording eye movement data during trial preparation, before the stimulus is shown on the screen.
2. SMITE.setBegazeTrialImage(). Send this image as soon as possible after the stimulus has been shown (e.g., directly after issuing the Flip command in PsychToolbox or PsychoPy). Import of eye movement data to BeGaze is started from this point, and all data recorded for this trial before the stimulus message are discarded by BeGaze but remain in the .idf file.
3. SMITE.stopRecording(). Stop the recording as soon as the trial has ended (e.g., when the stimulus is removed from the screen), ensuring that only relevant data are recorded and imported for analysis.

SMITE implements two further functions that place specially formatted messages in the .idf file that are understood by BeGaze. These functions allow for denoting mouse clicks and their location (SMITE.setBegazeMouseClick()), as well as key presses (SMITE.setBegazeKeyPress()). SMITE does not implement a method for automatically generating BeGaze XML files with AOI definitions, but the authors can provide guidance to anyone looking to implement this.

It should furthermore be noted that messages can only be sent to the .idf file using SMITE.sendMessage() when an active recording is in progress.

### Online gaze data

The underlying SMI iViewX SDK provides two ways of retrieving gaze data online: either through a function that reports the most recent data sample (available as SMITE.getLatestSample()) or as a stream handed to a user-provided callback function. To provide users with a convenient method of access to online data, SMITE has functionality to store the gaze data stream in a buffer by calling SMITE.startBuffer(). The data in the buffer can then be accessed using the functions SMITE.consumeBufferData() and SMITE.peekBufferData(), and buffering the gaze data stream can be stopped with a call to SMITE.stopBuffer(), which optionally also clears the buffer. SMITE.consumeBufferData() consumes the data in the buffer, emptying it, and SMITE.peekBufferData() leaves the returned data in the buffer so that it can be read multiple times. The SMITE.consumeBufferData() interface allows for returning only the *N* oldest samples in the buffer, but by default it returns the whole buffer. SMITE.peekBufferData() allows for getting the *N* newest samples in the buffer, but by default returns only the newest sample. Using this buffer interface, it becomes convenient to implement gaze-contingent paradigms in which it is important that no data samples be missed, such as when filtering the gaze signal (Špakov, 2012) or for some online event detection methods.

Using the buffer for recording data during an experiment may furthermore present a solution for users who no longer have access to BeGaze or the IDFConverter in the iTools package, and thus cannot access recordings stored in the proprietary .idf format anymore. Instead of saving eye movement recordings to these .idf files, such users could record the gaze data stream to the buffer during the whole experiment, and then save the buffer’s contents to an open file format of their choosing for later analysis. We advise that users exploring this use temporarily drain the buffer and store it to file during pauses in the experiment. Doing so during a trial may interfere with trial timing and is not recommended. If sufficient memory is available in the experiment machine, the user may also opt to drain the whole buffer to a file at once, at the end of the recording.

Two caveats should, however, be borne in mind when attempting this approach. First, not all the data fields in the .idf file are available in the streamed data samples. The contents of the streamed data samples are listed in Table 2, and notably they exclude (1) raw eye-tracking data—that is, the locations on the camera sensor of the pupil center and corneal reflections; (2) the 3-D gaze vectors provided by RED systems; and (3) the head position and orientation data provided by RED systems. Second, since the standard method of synchronizing the computer and eye-tracker times, by means of messages (SMITE.sendMessage()), is not available in this case, a routine would need to be implemented to synchronize the eye-tracker time to the timebase of the visual stimulation environment. This is required in order to be able to relate the eye-tracker data to events such as stimulus onsets. The authors can provide guidance to anyone looking to implement such synchronization. The authors have also started developing a MATLAB library to directly read the data stored in SMI’s proprietary .idf format. Although this may provide an alternative solution for users who can no longer convert .idf files, it should be noted that the code provided at https://github.com/dcnieho/SMIidfExtractor currently does not read all .idf files correctly.

**Table 2.** Contents of online data samples available through SMITE.getLatestSample(), SMITE.consumeBufferData(), and SMITE.peekBufferData()

Fieldname	Description
timestamp	Timestamp of sample
leftEye	Sample for the left eye
rightEye	Sample for the right eye
***Per eye:***	
gazeX	Horizontal gaze position on the screen
gazeY	Vertical gaze position on the screen
diam	Pupil diameter
eyePositionX	Horizontal eye position relative to the camera
eyePositionY	Vertical eye position relative to the camera
eyePositionZ	Distance to the camera

It should furthermore be noted that for iViewX-based eye trackers (the HiSpeed and first-generation RED systems) that run an older version of iViewX, the left and right eyes are swapped in the gaze data provided online. We have verified that this occurs for iViewX version 2.7.13, but that this bug no longer occurs in the last iViewX version, 2.8.43. To accommodate users stuck on these older iViewX versions, the online data provided through SMITE can be corrected using SMITE’s doFlipEye option, such that the data for a sample’s left eye correspond to the left eye of the subject. Note that this bug also affects the track status data available online through SMITE.rawSMI.getTrackingStatus(), where the values in the fields relativePositionX and positionRatingX need to be negated in order to correct the error. Finally, this bug also affects the eye images returned by the eye tracker (see the next section), which should be flipped horizontally such that the eye that is on the right side of the image corresponds to the right eye of the subject. The eye position in the track box and the eye image orientation are correct in SMITE’s setup interface (Figs. 1 and 2) if the doFlipEye option is set correctly. Data stored in the .idf file are not affected by this bug.

### Eye images

As in the case of gaze data, the underlying SMI iViewX SDK has a function that returns the latest eye image, and another functionality that provides a stream of eye images through a callback. We foresee two uses of eye images, either online access or storage to disk for later analysis. Since the first need likely exists for very few users, we have not implemented support for getting the latest eye image in SMITE. Users interested in this case can call SMITE.rawSMI.getEyeImage() in order to access the eye images online.^3^ To enable recording of eye images for offline storage, we have chosen to make use of a functionality that has been implemented by SMI in the iView eye-tracker server for all eye trackers except the RED250mobile and REDn. For the supported eye trackers, calling SMITE.startEyeImageRecording() starts the recording of eye images directly to disk, using the ET_EVB command that can be sent to the iView eye-tracker server. An advantage of this method is that images are also provided when track is lost, whereas in that case no images are provided by the online eye image retrieval functions for RED eye trackers. When track of the eyes is lost, the RED systems will store full-frame images to disk, instead of the cropped images of the eye region that are stored when the participant’s eyes are successfully tracked.

### Two-computer setups

A persistent problem with two-computer setups has been that when invoking the iV_saveData() call in the underlying SMI iViewX SDK, the .idf file is not stored on the system controlling the experiment but on the separate computer dedicated to running the eye tracker. Usually, it is instead desired for the data file to be stored on the computer running the experiment. When using SMI’s Experiment Suite to collect data, .idf files do appear on the experiment computer, but this is not possible to achieve with the SMI iViewX SDK. We have therefore analyzed how the SMI Experiment Suite achieves transfer of the .idf files from the eye-tracker computer to the experiment computer using Wireshark (www.wireshark.org), and have implemented the same method in SMITE.saveData(). For details, the interested reader is referred to the source code of this function.

### Direct access to SMI iViewX SDK

As we indicated above, SMITE only implements direct access to a subset of the functionality of the SMI iViewX SDK. The advanced user in need of niche functionality may wish to directly call the SMI iViewX SDK for full control over the eye tracker. When using SMITE, all functions of the SMI iViewX SDK can be accessed through the SMITE.rawSMI.<iViewX SDK function>() interface, as we demonstrated for acquiring eye images in the “Eye images” section above.

The MATLAB and Python interfaces to the SMI iViewX SDK that are distributed with SMITE, which can be accessed through the SMITE.rawSMI interface or can be used directly, have received several bug fixes and additions for completeness, as compared to the interfaces distributed with version 4.4.26 of the SMI iViewX SDK. We therefore recommend using our version of these interface functions even when not using SMITE. The files have furthermore been thoroughly documented for ease of use, and several convenience functions have been added to simplify use of the SMI iViewX SDK. For MATLAB, the interface is implemented in the files iViewXAPI.m, iViewXAPIHeader.m, SMIStructEnum.m, and SMIErrCode2String.m, and for Python in the files SMITE_raw.py, iViewXAPI.py, iViewXAPIReturnCodes.py, and helpers.py.

### MATLAB and Psychophysics Toolbox specifics

The MATLAB code supports both 32- and 64-bit versions of MATLAB (The MathWorks, Natick, MA). Because the 32-bit version of Psychophysics Toolbox was discontinued after version 3.0.11 in 2014, we recommend using the 64-bit version, to benefit from continued Psychophysics Toolbox development. The toolbox has been tested with 32-bit MATLAB R2015a and 64-bit MATLAB R2018a. Older versions of MATLAB might work but are untested. Note that Psychophysics Toolbox at the time of writing only supported MATLAB R2012a or later. All testing has been done on Windows 7. When using 64-bit MATLAB, it is important that Psychophysics Toolbox’s GStreamer dependency (type help GStreamer in the MATLAB command line) is correctly installed, for text rendering to work correctly on the setup screens.

Given that it is impossible to register MATLAB functions as callbacks with the SMI iViewX SDK, use of these callback functions is handled by a MATLAB extension (MEX) file, SMIbuffer. This MEX file is used by SMITE to record data into a buffer for online use. Direct access to the buffer object used by SMITE is possible using the property SMITE.rawBuffers. While buffering of online gaze data samples is handled through the SMITE functionality discussed in the “Online gaze data” section above, direct access to the SMIbuffer object enables receiving an online eye movement event stream, which is implemented in SMIbuffer but not exposed through SMITE. As was discussed in the “SDK features not implemented” section above, we recommend that the user carefully consider implementing their own online event detection method instead.

### Python and PsychoPy details

The Python version of SMITE only supports 32-bit Python, because PsychoPy is only available in a 32-bit version. SMITE has been tested with PsychoPy version 1.90.3 using Python 2.7, and PsychoPy version 3.0.2 using Python 3.6. Testing has been done on 64-bit Windows 7 machines.

## Example use and getting started

Two example programs are included in the SMITE distribution. The program named readme documents example uses of all functionalities of SMITE in a simple experiment that shows two trials consisting of a fixation point followed by an image while recording gaze data. The second program, named breakout, demonstrates using online data to enable the paddle in this game to be controlled through gaze. Full documentation is available in the readme.md file on the MATLAB and Python Github SMITE repositories.

SMITE is designed to make it easy to add eye-tracking functionality to existing experiments written in MATLAB/PsychToolbox or Python/PsychoPy. Follow the steps below to get started with SMITE.

1. Install the SMI iViewX SDK (version 4.4.26), on which SMITE depends.
2. Download SMITE from https://github.com/dcnieho/SMITE (MATLAB) or https://github.com/marcus-nystrom/SMITE (Python), and make sure it is placed on the MATLAB or Python path so that its functions can be located by the runtime environment. For Python, the command “pip install git+https://github.com/marcus-nystrom/SMITE.git#egg=SMITE will also install SMITE”.
3. Modify the readme script to interface with the SMI eye tracker you wish to use, and run it to verify that all is working correctly.
4. Copy over the relevant function calls from the readme script to your existing code or, alternatively, take the readme script as a skeleton and add your experiment code into it.

## Conclusion

SMITE, together with the PsychToolbox and PsychoPy platforms, provides a simple yet powerful way to develop eye movement research paradigms using SMI eye trackers. Given that SMI GmbH closed their business in summer 2017, and thus no longer supports their proprietary Experiment Suite solution for creating stimulus programs, we hope that SMITE offers users of SMI eye trackers a new route to keep using their machines.

Related to this are two further points of advice. First, users of an SMI eye tracker that is controlled through iViewX (these include the HiSpeed systems and the first-generation RED systems) should be very careful of the computer supplied by SMI that runs iViewX. iViewX is linked to the given computer and the hardware in it by a license key; it will not work when installed on another computer, and it will potentially stop working when components of the machine are changed. Since license keys are no longer provided by SMI, this means that if the computer running iViewX or a component in it breaks, the eye tracker can no longer be operated. It is thus imperative to keep the computer in good working order and to protect it from possible harm. Second, in at least one case, the last public release of SMI software had bugs that make the software inoperable. Specifically, the last version of the eye-tracker server software for the RED250mobile and REDn, iViewRED 4.4.26, has a bug that prevents calibration from succeeding, making it impossible to record data with the system. Users are therefore recommended to use the previous available version, iViewRED 4.4.11.

### Author note

We thank Richard Andersson and two anonymous reviewers for helpful comments on a previous version of the manuscript, and Katharina Scheiter, Juliane Richter, and Julius Meier from the TüdiLab for hospitality and help with testing during development of the toolbox. We gratefully acknowledge the Lund University Humanities Lab.

## Footnotes

1 In the MATLAB implementation, there is a single exception to this. Automatic file transfer on a two-computer setup (see section "Two-computer setups") relies on pnet, which is a library that comes with Psychophysics Toolbox but can also be downloaded separately from https://www.mathworks.com/matlabcentral/fileexchange/345-tcp-udp-ip-toolbox-2-0-6.

2 Note that all supported system except the REDn Professional provide eye images.

3 Note that to prevent a memory leak, it is recommended to use the input argument to SMITE.rawSMI.getEyeImage() to provide a single eye image struct (SMIStructEnum.Image) that is reused each time an image is requested.
